# Label-Free Single Cell Viability Assay Using Laser Interference Microscopy

**DOI:** 10.3390/biology10070590

**Published:** 2021-06-26

**Authors:** Yulia Beloglazova, Aleksandr Nikitiuk, Anna Voronina, Olga Gagarskikh, Yuriy Bayandin, Oleg Naimark, Victoria Grishko

**Affiliations:** 1 Perm Federal Scientific Centre, Institute of Technical Chemistry UB RAS, Academician Korolev St. 3, 614013 Perm, Russia; beloglazova.y@itcras.ru (Y.B.); voroninaao@gmail.com (A.V.); gagarsckih.olga@yandex.ru (O.G.); 2 Perm Federal Scientific Centre, Institute of Continuous Media Mechanics UB RAS, Academician Korolev St. 1, 614013 Perm, Russia; nas@icmm.ru (A.N.); buv@icmm.ru (Y.B.); naimark@icmm.ru (O.N.)

**Keywords:** cell viability assay, laser interference microscopy, non-invasive, MCF-7, Fourier analysis, wavelet analysis, multifractal analysis

## Abstract

**Simple Summary:**

Currently, only a few label-free methods for cell viability assessment are described in the literature. This paper covers a new label-free method based on the laser interference microscopy (LIM) to monitor the viability of single cells in real-time without dye incorporation. The metabolic activity of cells has been a key sign in assessing their viability with use of the LIM-aided method. On this basis, LIM allows the level of cell dynamics to be evaluated. We have analyzed the viability of attached and suspended cells by several spectral techniques of the LIM data processing and selected universal parameters for assessing their condition. Thus, LIM, as a highly sensitive quantitative phase imaging method, is applicable for assessing cell viability by a non-invasive mode combined with other cell assays.

**Abstract:**

Laser interference microscopy (LIM) is a promising label-free method for single-cell research applicable to cell viability assessment in the studies of mammalian cells. This paper describes the development of a sensitive and reproducible method for assessing cell viability using LIM. The method, based on associated signal processing techniques, has been developed as a result of real-time investigation in phase thickness fluctuations of viable and non-viable MCF-7 cells, reflecting the presence and absence of their metabolic activity. As evinced by the values of the variable *v_c_*, this variable determines the viability of a cell only in the attached state (*v_c_* exceeds 20 nm^2^ for viable attached cells). The critical value of the power spectrum slope *β_c_* of the phase thickness fluctuations equals 1.00 for attached MCF-7 cells and 0.71 for suspended cells. The slope of the phase fluctuations’ power spectrum for MCF-7 cells was determined to exceed the threshold value of *β_c_* for a living cell, otherwise the cell is dead. The results evince the power spectrum slope as the most appropriate indicator of cell viability, while the integrated evaluation criterion (*v_c_* and *β_c_* values) can be used to assay the viability of attached cells.

## 1. Introduction

The assessment of viability plays an important role in most mammalian cell-based biological, biomedical, and pharmacological studies [1]. In this case, viability means the physiological state of cells that allows carrying out their regular functions and ensuring tissue-specific activities and the possibility of cell division [2]. Viability determination is of specific importance for survival studies on immortalized cell cultures or cells from tissue samples affected by various factors, such as cultivation and cryopreservation conditions, investigation in apoptosis mechanisms in cells, as well as apoptotic cell death after exposure to cytotoxic drugs, etc.

Most extant methods for cell viability determination rely on assessing the morphological integrity of cells or their proliferative or metabolic activities [3,4]. To determine the number of viable cells, detection/visualization methods, such as bright-field and fluorescence microscopy, flow cytometry, and spectrophotometry by a plate reader, have often been used.

Bright-field microscopy is the simplest and most available observation method promoting the distinction viable cell in a specimen. Trypan blue (TB) is one of several dyes recommended for use as a stain to distinguish viable cells from dead cells by light microscopic quantitation. TB cannot penetrate the intact cell membrane of viable cells; by contrast, cells with damaged membranes are stained in a distinctive blue color readily observable under a microscope [5].

The spectrophotometric method has been widely used to assess cell viability by colorimetric evaluation of the metabolic activity of cells. The ability of cells to reduce tetrazolium salts, such as MTT (3-(4,5-dimethylthiazol-2-yl)-2,5-diphenyltetrazolium bromide), XTT (2,3-bis-(2-methoxy-4-nitro-5-sulfophenyl)-2H-tetrazolium-5-carboxanilide), etc., to colored formazan products only occurs in the mitochondria of metabolically active cells. Thus, color formation is a useful and convenient marker of viable cells. The quantity of formazan produced is proportional to the number of viable cells, which can be quantified by recording changes in absorbance using a plate-reading spectrophotometer [6].

Fluorescence-based assays are the most dominantly applied methods for exploring living cells. The approach focuses on two commonly used techniques: fluorescence microscopy and flow cytometry, allowing a fluorescent coloration to be detected by microscopic imaging or the assessment of the coloring intensity using a flow cytometer. In this case, fluorescent dyes are used, such as propidium iodide (PI), 7-aminoactinomycin D (7-AAD), ethidium bromide (EtBr), 4′,6-diamidino-2-phenylindole (DAPI), and SYTOX group dyes. The fluorescent probes selectively stain a cell with a damaged plasma membrane by interacting with DNA, while living intact cells are impermeable to these dyes. A more reliable method for evaluating cell viability using fluorescence microscopy is a combination of fluorescent membrane-permeable and -impermeable nuclear dyes, such as acridine orange and ethidium bromide, or Hoechst 33,342 and propidium iodide, respectively [7,8,9]. A similar approach based on a fluorescent double staining is traditionally used in the study of apoptosis by flow cytometry to accurately record the state of cells (early/late apoptosis, living/dead cells) [10,11,12]. In addition to the aforesaid, fluorescence microscopy is used frequently in combination with other imaging techniques. For example, confocal fluorescence microscopy can be used with atomic force microscopy [13,14], while intravital microscopy (IVM) technologies often combine different imaging methodologies, such as fluorescence-based assays, multiphoton microscopy, spinning disk microscopy, etc. Concurrently, during prolonged imaging, such methodologies are usually accompanied by the invasive effect of fluorescent vital probes on the research object [15,16].

Despite the extensive use, the aforementioned viability determination methods have also disadvantages, such as the requirement for various dyes, calibration and control materials for setting up devices and techniques and, especially, time-consuming sample preparation, including the steps of cell fixation and permeabilization, when dead cells losing their adhesive ability fall out of the cell population, thereby reducing the amount of dye staining in a sample [17]. DNA-binding dyes used for fluorescence microscopy can cause changes in the architecture of the nucleus and affect its structure and function, which leads to an inadequate reflection of the real situation in cells [18]. Furthermore, during prolonged cell imaging experiments, the fluorescence intensity markedly weakens by reason of photo-bleaching and can therefore give negative or defective results. A more detailed description of the advantages and disadvantages of modern cell viability assays can be found in recent reviews [19,20]. Thus, using known invasive methods gives no possibility to adequately assess the real viability and functional activity of cells, as well as to employ viable cells for further molecular genetic and biomedical studies [21,22].

Currently, the development of new real-time approaches to dynamic assessing cell viability without dye incorporation is of particular interest, since the monitoring of both cell functions and cell-to-cell communication in the cellular environment has enormous implications for cell biology and regenerative medicine [23]. Recently, non-destructive and label-free approaches for measuring the number of viable cells in real-time using a dielectrophoresis technique [24] and a luminescence luciferase-based assay [25] were developed. Laser interference microscopy (LIM) can be considered as a promising method allowing single cells to be explored without staining in real-time and dynamically. Typically, the LIM data contain information on local thickness and refractive index of the cell measurable structure, that allows for the analysis of morphometric properties of cells [26]. In addition, LIM as a highly sensitive quantitative phase imaging method, provides the real-time monitoring of dynamic processes of a single cell with high spatial resolution [27,28,29,30,31]. In principle, an adequate mathematical processing the LIM signals can ensure simultaneous or sequential use of several cellular research methods without prejudice to the test cell.

Previously, we developed a LIM-aided method enabling the morphometry and dynamics determination of viable cancer and non-cancer cells without any dyes [32,33]. In this method, the dynamics of the phase thickness of a single cell was recorded in real-time, near to the vicinity of the nucleolus boundary and considered as an integrating signal of cellular metabolic activity. Recently [34,35], the coherent dynamics of DNA open states was suggested to qualitatively characterize different scenarios of DNA gene expression for normal and cancer cells, with transitions among them being caused by various critical events. Generally, the changes occurring on the DNA scale, especially the critical events bringing a system out of the equilibrium state, correlate with the ones in the metabolic activity of cells. Therefore, as is the case with DNA, the metabolic activity level of cells can be evaluated by the analysis of the spatio-temporal scale invariants (in particular, power spectrum characteristics) of the optical thickness fluctuations of a cell [36].

Herein, we present a possibility of using the quantitative LIM data of cellular dynamics and a method for processing these data for a label-free single cell viability assessment.

## 2. Materials and Methods

### 2.1. Cell Line and Culture Conditions

An estrogen-dependent human breast adenocarcinoma cell line (MCF-7) was obtained from the N.N. Blokhin National Medical Research Center of Oncology (the Ministry of Health of the Russian Federation, Moscow). MCF-7 cells were maintained in Dulbecco’s Modified Eagle’s Medium – DMEM (PanEco, Moscow, Russia) with 10% fetal bovine serum (PAA Laboratories, Pasching, Austria), 2 mM L-glutamine (PanEco, Russia), and 1% penicillin/streptomycin (1000 U/mL; 10 mg/mL) (PanEco, Russia). MCF-7 cells were cultured in an Isotemp Barnstead CO_2_ incubator (Thermo Fisher Scientific, Waltham, MA, USA) under the following conditions: +37.0 ± 1.0 °C and 5.0 ± 0.5% CO_2_.

### 2.2. Cell Sample Preparation

#### 2.2.1. Suspension of Viable Cells

MCF-7 cells were cultured in a CO_2_ incubator for 48 h; then, the incubated cells were removed from the flask surface using a solution of 0.25% Trypsin (MP Biomedicals, Santa Ana, CA, USA)/Versene (0.2% EDTA in phosphate buffer, PanEco, Russia) (1:4). Next, the cells were pelleted and washed in 0.01M phosphate-buffered saline (PBS; PanEco, Russia) by centrifugation (at 150 G, 5 min, +37.0 ± 1.0 °C) on a Z 216 MK microcentrifuge (Hermle Labortechnik GmbH, Wehingen, Germany). After removal of the supernatant, the cell pellet was resuspended in 300 μL of PBS. A suspension of viable cells at a concentration 5 × 10^4^ cells/mL was therewith obtained.

The samples intended for imaging were prepared by translocating the suspension of viable cells into a zone restricted by a thin layer of silicone grease on the reflecting surface of a mirror slide; then, the zone containing the cells was covered with a coverslip, whereupon the samples were immediately examined by LIM.

#### 2.2.2. Suspension of Non-Viable Cells

MCF-7 cells were grown in the flask and harvested as described in Section 2.2.1. The obtained cell pellet was treated with a 4% paraformaldehyde solution (PFA) in PBS for 20 min at +4 ± 1.0 °C, while stirred in a Vortex mixer (model V1-Plus, Biosan, Riga, Latvia). Next, the suspension of non-viable cells was washed twice with 0.01M PBS, and the sediment was resuspended in 300 μL of PBS (5 × 10^4^ cells/mL).

The samples for imaging were prepared by translocating the suspension of non-viable cells as described in Section 2.2.1.

#### 2.2.3. Viable Cells Attached to a Glass Surface

MCF-7 cells were seeded into a 60 mm Petri dish with a glass coverslip 24 × 50 mm^2^ at the bottom at a density of 2 × 10^4^ cells/mL and cultured for 48 h in a CO_2_ incubator as described above. 48 h later, the samples for imaging were prepared by translocating the coverslip with attached viable cells as described in Section 2.2.1.

#### 2.2.4. Non-Viable Cells Attached to a Glass Surface

MCF-7 cells (2 × 10^4^ cells/mL) were seeded into a 60 mm Petri dish containing a glass coverslip and incubated for 48 h. Non-viable cells attached to the glass surface were obtained by fixation of the cells using a 4% PFA solution for 20 min at +4 ± 1.0 °C. The formaldehyde-fixed cells were washed twice with 0.01M PBS (PanEco, Russia). The coverslip with the attached non-viable cells was translocated onto the silicone zone of a slide and then visualized by LIM as described in Section 2.2.1.

### 2.3. Trypan Blue Staining

The Trypan Blue exclusion method was used to corroborate the correctness of the cell sample preparation methods: a portion of the obtained cell suspensions and one of cells attached to the glass surface were stained in parallel with 0.4% Trypan Blue for 5 min at room temperature. The cells incorporating the dye (non-viable) or the ones excluding the dye (viable) were counted in a routine manner using a dual-chamber hemocytometer and an optical microscope.

Another unstained portion of the cells was analyzed by the LIM method.

### 2.4. Cell Research by LIM

#### 2.4.1. Technical Characteristics of the Laser Interference Microscope MIM-340

The laser interference microscopy (LIM) is based on the determination of local phase shifts of the light wave modulated by a cell. To estimate the dynamics of cells, their optical (phase) thickness was measured using a laser modulation interference microscope, model MIM-340 (Shvabe, Moscow, Russia). A semiconductor laser with 655 nm wavelength was used as a coherent radiation source. Vertical and lateral resolutions equaled 0.3 and 100 nm, respectively. All the phase images were collected at ×4050 magnification. The frequency record of the optical thickness fluctuations of the cells registered by the MIM-340 microscope equaled 33 Hz. The microscope kit includes a granite base plate designed to ensure temperature stability, which, essentially, is an anti-vibration table for placing samples.

#### 2.4.2. MIM-340 Laser Measurement of Phase Thickness Fluctuations

**Figure 1:**
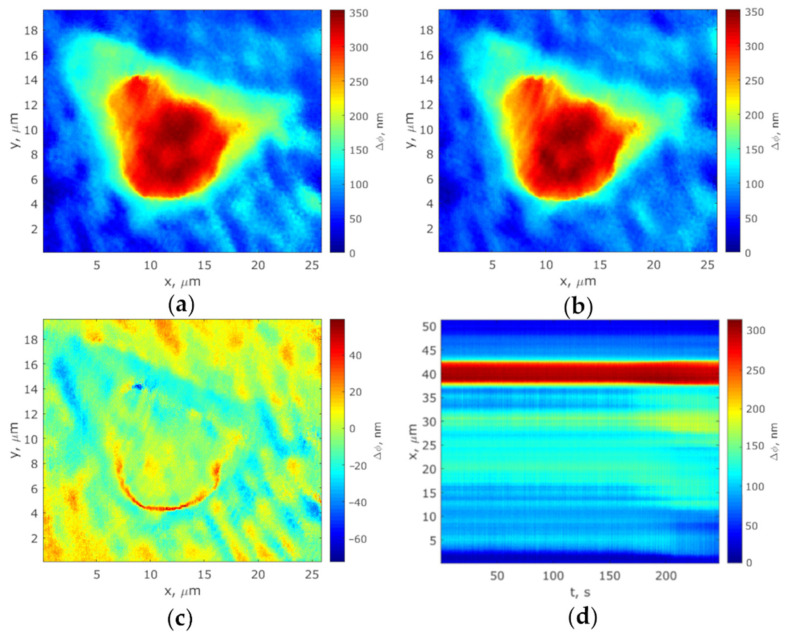
Protocol of scan line determination and registration of the phase thickness cell profile of a cell: (**a**) Frame Nr.1; (**b**) Frame Nr.2; (**c**) Difference frame; (**d**) Track diagram. Red and deep red colors correspond to nucleus and nucleolus of the cell.

The phase thickness fluctuations were registered at room temperature and humidity according to the following algorithm. A cell phase image (frame Nr.1) was taken. Half a minute later, a new cell phase image (frame Nr.2) was taken. Then, the “frame Nr.2—frame Nr.1” difference frame was calculated, and the position of the scan line was determined, along which the laser microscope records the phase thickness profile. Next, the fluctuations Δ*ϕ* (*t*,*x*) of the phase thickness of a cell along the setting scan line were measured for 247 s and saved as a track diagram (Figure 1).

#### 2.4.3. Phase Thickness Fluctuations Analysis

The raw, unfiltered data were analyzed using an original algorithm implemented in the MathWorks Matlab software. The algorithm was designed to analyze the signals of the phase thickness fluctuations of a cell, to filter the utmost fluctuating signal (intense fluctuations are usually observed in steep sections of the phase thickness profile [29]) and calculate the variance of the signal. Filtration was made by the one-dimensional continuous wavelet transform method. The Morlet wavelet was used as a basic wavelet, with all the low-level noises filtered out.

The extracted signal as a function of time and the corresponding dynamic parameters such as a variance, slopes of power spectrum and integral wavelet spectrum, global Holder exponent, Hurst exponent, and width of multifractal (singularity) spectrum were used to characterize the fluctuations.

The variance *v* was determined according to the following equation:

(1) $$v=\frac{1}{N-1}\sum_{i=1}^{N}{\left|\Delta \phi \left(t_{i}\right)-\mu \right|}^{2},$$

where ∆*ϕ*(*t_i_*) is the phase thickness fluctuations at instant *t_i_, N* is the number of phase thickness fluctuations of a cell, *μ* is the mean value of ∆*ϕ(t).*

The slope of power spectrum *β* was calculated as

(2) $$\hat{f}\left(k\right)=\frac{1}{\sqrt{2\pi }}\int_{-\infty }^{\infty }e^{-itk}\Delta \phi \left(t\right)dt,$$

(3) $$S\left(k\right)={\left|\hat{f}\left(k\right)\right|}^{2},$$

(4) $$S\left(k\right)\frac{1}{k^{\beta }},$$

where $\hat{f}\left(k\right)$ is the Fourier transform of ∆*ϕ(t)*, *k* is the frequency, *S(k)* is the power spectrum.

The slope of integral wavelet spectrum *γ* is described by a one-dimensional continuous wavelet transform having the following form:

(5) $$W\left(k,b\right)=\int_{-\infty }^{\infty }{\left|k\right|}^{-1/2}\psi {\left(\frac{t-b}{k}\right)}^{}\Delta \phi \left(t\right)dt,$$

(6) $$E\left(k\right)=\frac{1}{k}\underset{b}{\sum }{\left|W\left(k,b\right)\right|}^{2},$$

(7) $$E\left(k\right)\frac{1}{k^{\gamma }},$$

where *b* is the shift, *ψ* is a basic wavelet (the Morlet wavelet), *W(k,b)* are wavelet coefficients, *E(k)* is the integral wavelet spectrum. In Equation (6) the first multiplier is a norm of the integral wavelet spectrum and is required for comparison with the power spectrum.

The global Holder exponent *α_global_*, the Hurst exponent *H*, and the width of multifractal spectrum ∆*α* are given by:

(8) $$Z\left(q,a\right)=\sum_{l\in L\left(a\right)}{\left(a\geq a^{\prime }\left|W\left(a^{\prime },t_{l}\right)\right|\right)}^{q},$$

(9) $$Z\left(q,a\right)a^{\tau \left(q\right)},$$

(10) $$\{\begin{array}{c} \alpha =\partial \tau \left(q\right)/\partial q,\\ f\left(\alpha \right)=\underset{q}{min}\left(q\alpha -\tau \left(q\right)\right), \end{array}$$

(11) $$\alpha_{global}={\alpha \;}_{f_{max}},$$

(12) H=α(q=2),

(13) $$\Delta \alpha =\alpha min_{max},$$

where *a* is the scale, *q* indicates real numbers (ranged from −2 to 2), *L(a)* is a set of lines of local extremes (*l*) on the scale *a, t_l_* characterizes the position on the scale a of the extremum related to the line l, *a’* is a scale reduced as compared with the specified scale *a, τ(q)* are scaling exponents, *α* are Holder exponents, *f(α)* is the singularity spectrum. Equations (8)–(10) in block represent the wavelet transform maxima modulus method employed for multifractal analysis [37]. The ratio (8) means the maximum value of the local extremum modulus (wavelet transform coefficients) selected along each line on the scales as smaller than the set value *a*. The scaling exponents were determined according to the ratio (9) at *a* → 0. The system (10) is termed the Legendre transform.

#### 2.4.4. Statistical Analysis

To statistically analyze the dynamic parameters of the independent samples of the viable and non-viable cells, the two-sample non-parametric Wilcoxon–Mann–Whitney test was used. During statistical processing, the median, the 25th and 75th percentiles, and confident intervals (with most extreme points not being considered as outliers) were determined. The processed results were statistically analyzed with the use of the MathWorks Matlab software.

The statistical processing of the experimental data obtained in three independent cellular experiments reiterated thrice was carried out by determining the mean values and their standard deviations (SD).

## 3. Results and Discussion

MCF-7 cells are commonly used adherent breast cancer cells isolated from the pleural effusion of a 69-year-old woman with metastatic disease in 1973 by Dr. Soule and colleagues at the Michigan Cancer Foundation [38,39]. MCF-7 cells have been used as a model in cell and molecular biology experiments, fundamentally impacting breast cancer research [40,41,42]. Antecedently, we used attached native MCF-7 cells and MCF-7 cells under the action of pro-apoptotic agents to evaluate morphometric parameters recorded by LIM [32]. In this work, we investigated the viability of MCF-7 cells obtained in the suspended and attached state to mimic various experimental conditions. The state of cells plays an important role in the functioning of the human body and affects the features of a cell research protocol. For example, cell adhesion is crucial for normal embryonic development and adult maintenance of multi-cellular organisms by regulating cell migration, tissue organization, immune responses, and wound healing [43]. Thus, we evaluated the possibility of using both suspended MCF-7 cells and MCF-7 cells attached to the glass surface of a coverslip to determine their viability by the LIM method.

**Figure 2:**
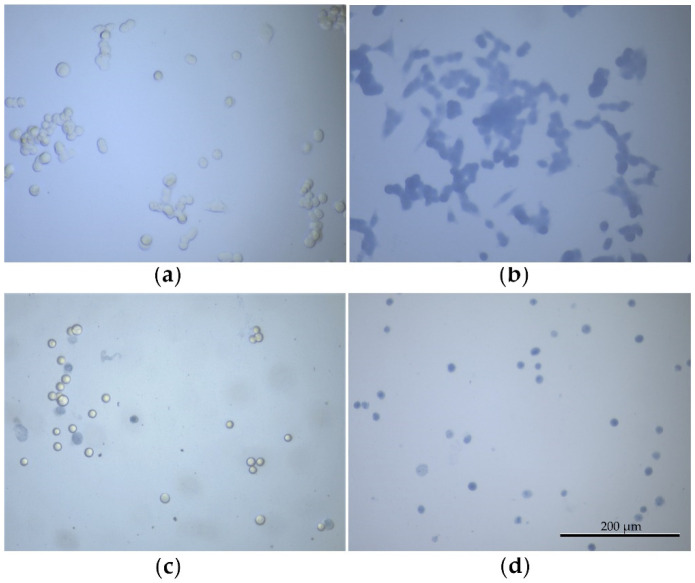
Trypan Blue staining: (**a**) viable cells attached to the glass surface; (**b**) non-viable cells attached to the glass surface; (**c**) suspended viable cells; (**d**) suspended non-viable cells. The images were obtained in the navigation channel of the MIM-340 microscope at ×810 magnification. Scale bar in the figure represents 200 μm.

To corroborate the correctness of the cell sample preparation methods, the viability of MCF-7 cells was quantitatively assessed by Trypan Blue (TB) staining (Figure 2). According to TB, the viability of attached and suspended viable cells was 98.11 ± 0.42 and 94.36 ± 0.35%, respectively (the data are expressed as the mean ± SD). The slightly declined viability of suspended viable cells may have been caused by a mechanical impact during centrifugation and washing of the cells. In contrast, both the suspended and the attached cells after fixation were labeled by the TB as non-viable cells (the viability of such samples equaled 0.00%).

The non-invasive high-resolution imaging of living cells in their natural ambience, without exogenous contrast or labels being used, is a prerequisite for the accurate visualization of biological processes at the cellular and molecular levels [44]. For example, recent advances in real-time analysis of the dynamics and changes of cellular activity by quantitative phase imaging (QPI) techniques have shown promises for the cellular-level understanding of the pathophysiology of diseases [45]. The concepts developed in a recent review [35] concern the metabolic activity of cells, including pathological transformations and their viability as metabolic processes in substantially non-equilibrium thermodynamic systems. A feature of these processes is the “subordination” of the evolution of biological systems to universal (self-similar) scenarios, when morphogenetic processes are determined by collective (mesoscopic) variables. The dynamics of these variables, associated with open complexes, significantly depends on the thermodynamic potential of the system (associated with the epigenetic landscape), which determines the structural transitions in cells, including their critical dynamics during pathological transformations. In this case, it is of fundamental importance to identify the functional type of thermodynamic potential on open complexes, whose dynamics correlates with the phase thickness fluctuations of the cells investigated by LIM. Thus, the dynamic parameters of the phase thickness fluctuations mirror information on the metabolic activity of cells and can therefore be used to evaluate their viability.

A new method for LIM-aided cell viability assessment developed by us has ensued from investigating the cell phase thickness fluctuations reflecting cellular real-time dynamics. The applicability of mathematical methods for signal processing so to assess cell viability was investigated on the basis of the analysis of dynamic parameters obtained in this processing. The dynamic parameters of viable and non-viable cells were measured by the processing of phase thickness fluctuations Δ*ϕ*(*t*,*x*) of a single cell using the MIM-340 microscope. Total data for 307 MCF-7 cells were obtained using the LIM method, including 162 viable (86 attached and 76 suspended cells) and 145 non-viable cells (86 attached and 59 suspended cells).

**Figure 3:**
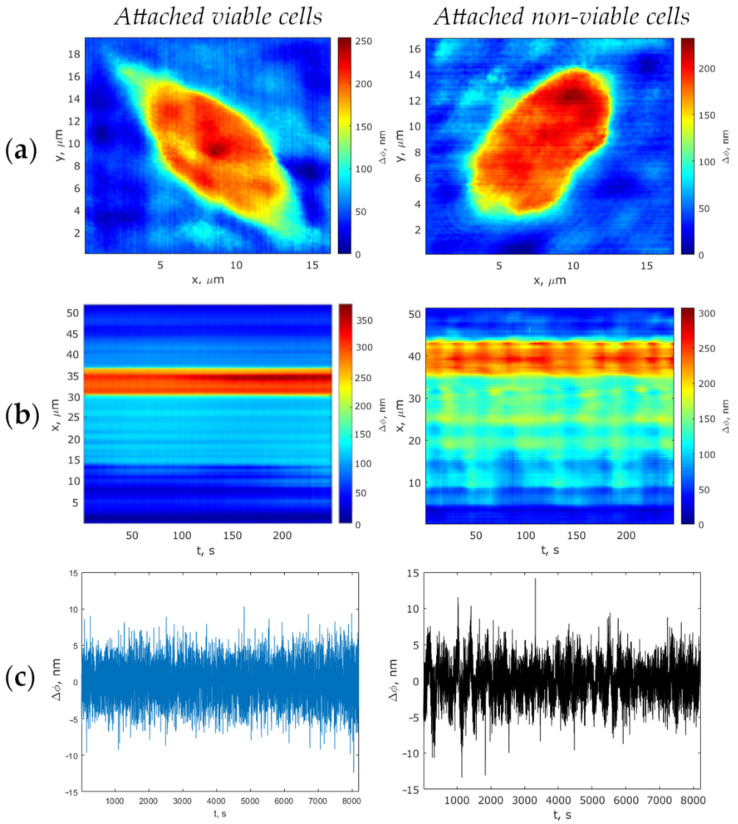
Comparison of viable and non-viable cells attached to the substrate: (**a**) Phase images; (**b**) Track diagrams; (**c**) Phase thickness fluctuations of the cells. In the x-direction, spatial coordinates (**a**), and time (**b**,**c**) are positioned; in the y-direction, spatial coordinate (**a**,**b**) and phase thickness (**c**) are positioned.

**Figure 4:**
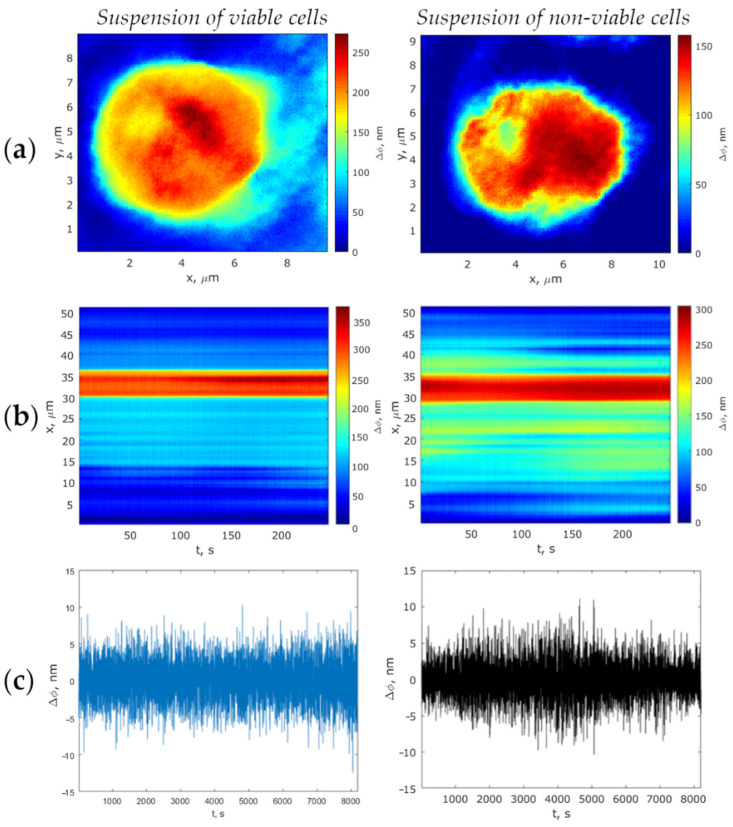
Comparison of viable and non-viable suspended cells: (**a**) Phase images; (**b**) Track diagrams; (**c**) Phase thickness fluctuations of the cells. In the x-direction, spatial coordinates (**a**), and time (**b**,**c**) are positioned; in the y-direction, spatial coordinate (**a**,**b**) and phase thickness (**c**) are positioned.

The phase images of viable and non-viable attached and suspended MCF-7 cells graded on a pseudo-color scale according to the phase thickness of their subcellular structures are presented in Figure 3a and Figure 4a. As is apparent, both cell boundary and cellular constituents, such as cytoplasm, nucleus, and nucleolus, are distinctly discernible in the phase images, with the nucleolus being discernible as an optically denser zone.

Track diagrams containing information on changes in the cell profiles during the measurement of the optical thickness by the LIM method, were plotted as per the algorithm described in Section 2.4.2. The corresponding phase thickness fluctuations of viable and non-viable cells presented as typical track diagrams are shown in Figure 3b and Figure 4b (for suspended cells and those attached to the glass surface, respectively). On the ground of the fact that the metabolic activity of cells is most noticeably manifested by a local enhancement of the fluctuation intensity in the vicinity of the nucleolus boundary [31], the measurement of the dynamic parameters of phase thickness fluctuations in our study was recorded along the scanline of the topogram passing through the nuclear and in the nucleolus region (deep red zone). Therefore, the diagrams show evidence of one-dimensional signals corresponding to the most intensive fluctuating area of a cell. Figure 3c and Figure 4c show the living cells’ fluctuations as being more intense compared with the phase thickness fluctuations of dead cells.

Recently [46], the movements of prokaryotic and eukaryotic samples were recorded and experimentally measured at a subnanometer resolution by the cantilever of an atomic-force microscope, and living biological objects were shown to differ from non-living ones in large amplitudes of physical values’ fluctuations. The nanomotion signal generated by the cell is composed of vibrations arising from many metabolically related sources that combine energy consumption with local movement or molecule redistribution [47]. Thus, the intensity of the cantilever movements directly correlates with the viability of samples and their metabolic activity. Moreover, live cancer cells with the best viability induced a larger fluctuation of the microcantilever [48].

There are many studies [49,50,51,52] of the spectral (in terms of the Fourier and wavelet analysis) and multifractal properties of a biological system dynamics. Particularly, a recent paper [35] showed that the dynamics of living breast human cells in cancerous and normal states differ according to multifractal properties. Cancer cells evince monofractal dynamics, while normal cells are characterized by multifractal spectra. With these results as a basis, the multifractal analysis technique can also be applicable to identifying differences between viable and non-viable cells.

Therefore, in accordance with previous studies [46,47,48], the variable *v* was chosen by us as a parameter of phase thickness fluctuations of viable and non-viable cells recorded using the LIM. Further, we estimated the power and integral wavelet spectra, in particular, the slopes of the power spectrum *β* and the integral wavelet spectrum *γ*. After that, the multifractal spectra were compared, namely, the global Holder exponent *α_global_*, the Hurst exponent *H*, and the width of multifractal spectrum ∆*α*. Phase thickness fluctuations of the LIM data were analyzed according to the algorithms described in Section 2.4.3.

In the first stage of the cell dynamic data processing, the variables of viable and non-viable attached cells’ samples (samples Nr.1 and Nr.2, respectively) were comparatively analyzed. For the viable cells, the median values of the variable equaled approximately 66.4 nm^2^. For the non-viable cells, the variable fell to a quite low value 15.3 nm^2^, while the median values of the variables of viable and non-viable suspended cells (samples Nr.3 and Nr.4, respectively) practically did not vary: ~11.5 nm^2^ and 9.7 nm^2^, respectively. According to the Wilcoxon–Mann–Whitney criterion, the zone of intersecting values between samples Nr.1, Nr.2 and Nr.3, Nr.4 was determined as being small enough and corresponding to a *p*-value < 0.001 for the attached cells and a *p*-value < 0.001 for the suspended cells. To determine the critical value of the variable *v_c_* of the phase thickness fluctuations, relative to which the cell viability can be judged from the LIM data, the 25th and 75th percentiles values of the samples were estimated (Table 1). With these estimates as a basis (with the 75th percentile value equaling 20.6 nm^2^ for non-viable attached cells, and the 25th percentile value equaling 21.2 nm^2^ for viable attached cell), we figured out that the variable of the phase thickness fluctuations of a cell can determine cell viability only in the case of a cell attached to the substrate. At this point, the variable of the phase thickness fluctuations for viable attached cells (*v_c_*) was found to approximately exceed 20 nm^2^. Thus, at this stage, in the case of the phase thickness fluctuations’ variable of an attached cell established as exceeding 20 nm^2^, the cell may be regarded as viable.

In the second stage of the study, the samples Nr.1 and Nr.3, as well as the samples Nr.2 and Nr.4 were comparatively analyzed (Table 1). The Wilcoxon–Mann–Whitney test revealed a significant difference between the first two samples (*p* < 0.001), with the latter ones found as not differing (*p* > 0.05). Thus, non-viable cells show similar dynamics attributed to the absence of their metabolic activity in both the suspended and the attached state. Concurrently, the viable cells (as dependent on their state, i.e., adhesion or suspension) differ from each other on the level of phase thickness fluctuations.

**Figure 5:**
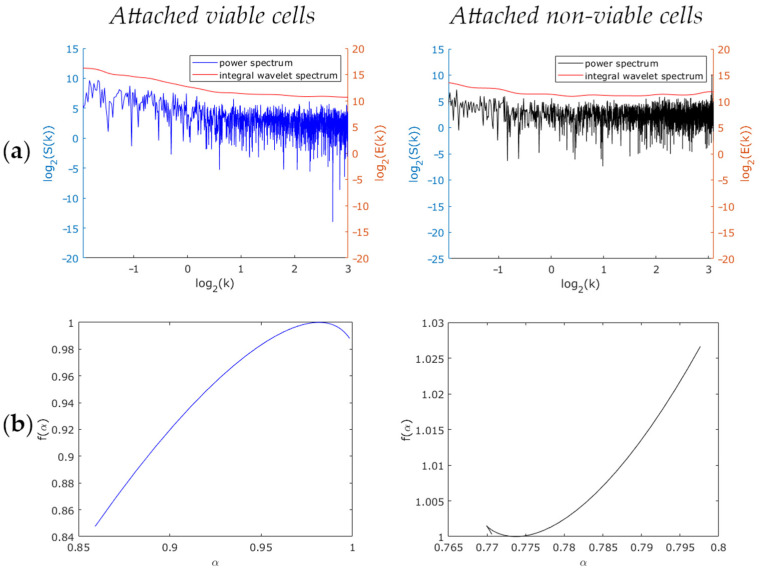
Comparison of viable and non-viable cells attached to the glass surface: (**a**) Power spectrum and integral wavelet spectrum; (**b**) Multifractal spectra.

**Figure 6:**
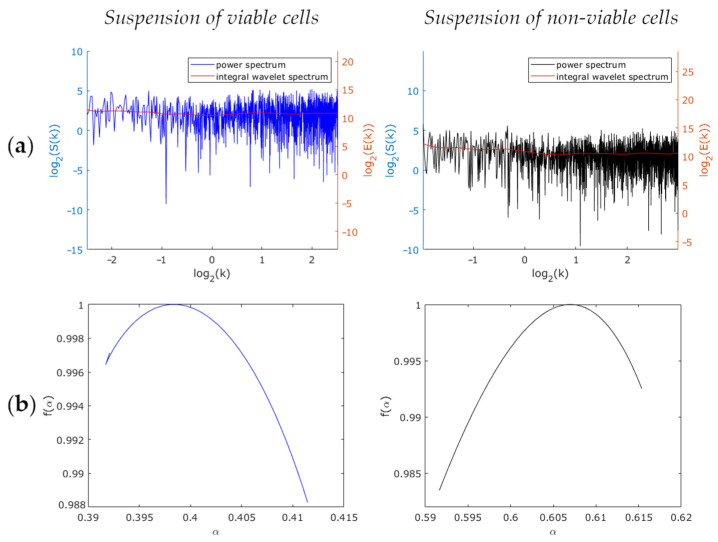
Comparison of viable and non-viable suspended cells: (**a**) Power spectrum and integral wavelet spectrum; (**b**) Multifractal spectra.

**Figure 7:**
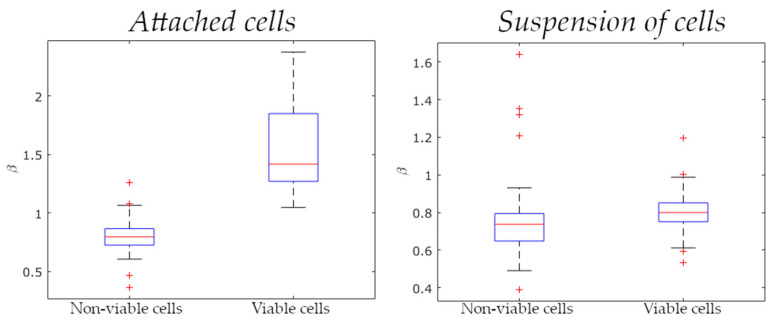
Power spectrum slopes of optical thickness fluctuations for the attached and suspended viable and non-viable MCF-7 cells (*p* < 0.001). In each box, the central mark denotes the median, and the bottom and top sides of the box denote the 25th and 75th percentiles, respectively. The whiskers extend to the most extreme points not considered as outliers, with the outliers plotted individually using the “+” symbol.

Next, the samples Nr.1, Nr.2 and the samples Nr.3, Nr.4 were comparatively analyzed as to the slope of the Fourier power spectrum and that of the integral wavelet spectrum of a filtered signal with the highest variable of optical thickness fluctuations (see typical spectra in Figure 5a and Figure 6a, respectively). The power spectrum slopes and the integral wavelet spectrum having—according to the results of Fourier and wavelet analysis—the same value, we therefore focused only on the power spectrum slope (*β*) parameter. We found that in the case of viable and non-viable attached cells, the median values of the power spectrum slopes differed approximately twofold (Figure 7). For viable cells, these values equaled approximately 1.4 (sample Nr.1) and 0.8 (sample Nr.3), for non-viable cells ~0.8 (sample Nr.2) and 0.6 (sample Nr.4). According to the Wilcoxon–Mann–Whitney criterion, the zone of intersecting values between the attached and the suspended cell samples was found as being distinctly different (*p* < 0.001). To determine the critical value of the power spectrum slope of *β_c_* fluctuations of optical thickness, relative to which cell viability, according to the LIM data, may possibly be judged, the values of samples corresponding to the 25th and 75th percentiles were estimated (Figure 7). Based on these estimates, the conclusion was drawn that the value of *β_c_* equals 1.00 for attached MCF-7 cells and 0.71 for suspended MCF-7 cells.

Further, we comparatively analyzed also the samples of the Fourier power spectrum slope of filtered-out signals with the highest variable of the optical thickness fluctuations of the attached and suspended viable cells, as well as of non-viable cells. The Wilcoxon–Mann–Whitney test ascertained a marked difference between the first two samples (*p* < 0.001) and between the latter ones (*p* < 0.01). In both cases, viable and non-viable cells are meant to differ in terms of the power spectrum slope.

According to the multifractal analysis results (see typical spectra in Figure 5b and Figure 6b, respectively), the parameters (width, global Holder, and Hurst exponents) of the singularity spectrum of the suspension of viable and non-viable cells were found not to differ from each other (*p* equals 0.44), while those of the attached cells differed (*p* < 0.001). In all cases, except for viable attached cells, the spectra turned out to be monofractal. The median width of the multifractal spectra of viable attached cells equaled 0.8. With these results as a basis, we concluded that multifractal analysis enables the evaluation of the viability of attached cells only.

As the noise component (the movement of a free cell in a nutrient medium) markedly contributes to cellular dynamics, the optical thickness fluctuation values of viable and non-viable suspended cells measured and processed most often differ only slightly. As a result, only the power spectrum slope (or the integral wavelet spectrum) appears to be a more universal indicator reflecting the inherent links of cellular metabolic activity (viability). The criterion based on this evaluated parameter is formulated as follows: if the power spectrum slope of the phase fluctuations of MCF-7 cells exceeds the threshold value of *β_c_*, this cell is alive, otherwise it is dead (irrespectively of whether the cell is attached to the substrate surface or suspended).

## 4. Conclusions

To monitor the viability of cells in the suspended and attached (cell adhesion) states in real-time and by a non-invasive mode, we described a new label-free LIM-based method that can be multiplexed with other cell assays. The algorithm, developed to assess cell viability as applied to MCF-7 cells, relates to the dynamics of a single cell (fluctuations in the phase thickness of cells) and can be used to explore the viability criterion for eukaryotic cells of different types.

Our results evinced differences in the viability of MCF-7 cells attached to a glass surface and of those in suspension. The processed dynamic data for viable and non-viable cells allowed determining a critical value of the variable (*v_c_* ≈ 20 nm^2^), provided that the viability was evaluated only for attached cells. Concurrently, the slope of the power spectrum *β_c_* was selected as a more universal parameter to assess the state of a cell and therewith identify viable and non-viable attached and suspended cells. The conclusion was drawn that for attached MCF-7 cells, the *β_c_* value equals 1.00, while for suspended MCF-7 cells, this value equals 0.71. Thus, to investigate the viability of attached cells, an integrated evaluation criterion can be used (the variable and the power spectrum slope, conjointly). In contrast, in the case of suspended cells, only the method based on the evaluation of the power spectrum slope of the phase fluctuations is applicable.

As with other non-destructive and label-free approaches for measuring the number of viable cell in real-time [24,25], the LIM method is based on the metabolic activity of cells. Therefore, it is recommended that the power spectrum slope of the phase fluctuations be empirically determined for each cell type.

It should be noted that the development of microscopic methods for studying adherent cells is of special relevance to cell biology and biomedical research: adhesion playing a central role in such biological processes as invasion and metastasis of cancer cells, stem-cell fate, cell death, and/or growth arrest. The detachment of adherent cells or the loss of their ability to adhere can be used as a marker of cell death in a cytotoxicity test or as a marker of cell detachment-induced apoptosis [53]. The microscopy of cellular adhesion is also essential for a more comprehensive understanding of cell–substrate interactions [54,55]. This understanding is of paramount importance for the development of neoteric drugs to facilitate or inhibit cell–substrate interactions. Such drugs can include agents favoring cell adhesion, in case of tissue regeneration or biomaterial integration, or inhibiting it, in case of metastasis or fibrosis [56]. Thus, the LIM study of the metabolic activity of cells and the proposed signal processing techniques of cellular dynamics may be useful not only for assessing cell viability, but also for studying various functional states of cells [57,58,59].

## 5. Patents

An application “Method for determining the viability of eukaryotic cells by laser interference microscopy” is pending at the Eurasian Patent Organization (EAPO) (application number—202000269).

An application for registration of the computer program “Program for the assessment of cell viability’’ has been submitted to the Federal Institute of Industrial Property (FIPS) (application number—2020618535).

## Figures and Tables

**Table 1:** Estimation of the variables’ values of the 25th and 75th percentiles of viable and non-viable MCF-7cell samples.

Percentiles	Attached Viable Cells (Sample Nr.1)	Attached Non-Viable Cells (Sample Nr.2)	Suspended Viable Cells (Sample Nr.3)	Suspended Non-Viable Cells (Sample Nr.4)
25th	21.2	9.9	7.0	7.6
75th	111.6	20.6	16.0	12.8

## Data Availability

Not applicable.
